# Supplementary material for: Effect of TRPV4 Antagonist GSK2798745 on Chlorine Gas-Induced Acute Lung Injury in a Swine Model
Source: Int J Mol Sci. 2024 Apr 2;25(7):3949. doi: 10.3390/ijms25073949 (PMC11011849; doi:10.3390/ijms25073949)
Supplement: Supplementary file 1 [file ijms-25-03949-s001.zip › ijms-2841001-supplementary.pdf]

Supplemental Materials

Supplemental Tables

Table S1. Model Development Study Animal Demographics and Exposure/Treatment Assignments

Table S2. Dose Response Study Animal Demographics and Exposure/Treatment Assignments

Supplemental Figures

Figure S1. Chlorine Exposure System Schematic

Figure S2. Kinetics of Functional Lung Injury during Chlorine Exposure

Figure S3. Kinetics of Functional Lung Injury following Chlorine Exposure

Figure S4. GSK2798745 Plasma Concentration vs. Time Profiles

Figure S5. Functional Lung Measurements Unaffected by GSK2798745 Treatment

Table S1. Model Development Study Animal Demographics and Exposure/Treatment Assignments

Animal ID	Sex	Approx. Age at Study Start (weeks)	Body Weight (kg)	Exposure Duration (min)	Targeted Cl_2 Dose (mg/kg)	Actual Cl_2 Dose (mg/kg)	GSK'745 Dose Group	Survival (hours)
7465	Female	13.0	30.2	60	0	0	N/A	24
7440	Male	12.7	30.4	60	0	0	N/A	24
7443	Male	18.4	37.3	19	2.60	2.09	N/A	24
8372	Female	14.4	29.0	30	4.10	3.00	N/A	24
8375	Male	15.7	28.2	40	5.47	4.04	N/A	24
8996	Male	11.3	31.6	45	6.16	4.36	N/A	24
8998	Female	11.3	30.4	45	6.16	4.79	N/A	24
8371	Female	16.7	33.6	50	6.84	5.20	N/A	24
8992	Male	14.9	28.8	50	6.84	5.83	N/A	24
8972	Female	13.1	30.2	50	6.84	4.77	N/A	24
8993	Male	17.0	34.4	55	7.52	6.27	N/A	24
8999	Female	15.4	33.2	55	7.52	5.91	N/A	24
1224	Male	13.3	26.2	55	7.52	6.08	N/A	24
1221	Female	14.4	25.8	55	7.52	5.67	N/A	24
1745	Male	11.4	29.6	55	7.52	5.15	N/A	24
1746	Female	11.5	25.6	55	7.52	4.82	N/A	24
8374	Male	13.0	28.2	60	8.21	5.27	N/A	<1
9917	Female	12.6	29.0	65	8.89	6.83	N/A	24
9914	Male	12.4	29.4	65	8.89	7.12	N/A	24
9918	Female	14.0	30.4	75	10.26	7.64	N/A	24
9913	Male	14.3	29.2	75	10.26	8.33	N/A	24
1219	Female	11.6	27.0	88	12.04	8.83	N/A	0
7458	Female	16.4	33.8	89	12.18	11.10	N/A	<1
1222	Male	11.6	28.2	90	12.31	9.81	N/A	24

Table S2. Dose Response Study Animal Demographics and Exposure/Treatment Assignments

Animal ID	Sex	Approx. Age at Study Start (weeks)	Body Weight (kg)	Exposure Duration (min)	Targeted Cl_2 Dose (mg/kg)	Actual Cl_2 Dose (mg/kg)	GSK'745 Dose (mg/kg)	Survival (hours)
2050	Female	28	40.7	55	0	0	0	24
2064	Female	31	51.7	55	7.52	5.84	0	24
2069	Male	29	38.2	55	7.52	5.63	0	24
2051	Female	29	44.4	55	7.52	5.52	0	24
2076	Male	30	50.5	55	0	0	0	24
2044	Female	31	44.7	55	0	0	0	24
2077	Male	31	39.4	55	7.52	4.91	0	24
2066	Female	35	55.4	55	7.52	5.99	0.0714	24
2079	Male	35	47.5	55	7.52	5.95	0.0006	24
2056	Female	36	47.3	55	7.52	6.11	5.94	24
2091	Male	35	47.7	55	7.52	5.93	0.0072	24
2052	Female	35	54.2	55	7.52	4.57	0.792	24
3408	Male	13	36.1	55	7.52	4.77	5.94	24
3398	Female	13	38.3	55	0	0	0	24
3404	Male	13	37.7	55	7.52	4.70	0.0072	24
3411	Male	15	35.1	55	0	0	0	24
3395	Female	15	35.2	55	7.52	4.67	0.0006	24
3400	Female	15	35.0	55	7.52	4.97	0.0714	24
3071	Male	15	37.0	55	7.52	4.57	5.94	24
3409	Male	16	36.0	55	7.52	4.66	0.792	24
3394	Female	16	38.2	55	7.52	4.69	5.94	24
3396	Female	16	34.5	55	7.52	4.95	0	24
3412	Male	16	29.7	55	7.52	4.90	0.0006	24
3401	Female	17	39.2	55	7.52	4.75	0	24
3406	Male	17	39.3	55	7.52	4.88	0.0714	24
3066	Male	17	36.1	55	7.52	4.94	0	24
3393	Female	17	40.7	55	7.52	4.84	5.94	24
3405	Male	18	44.0	55	7.52	4.69	0.0072	24
3060	Female	18	49.0	55	7.52	4.62	5.94	24
3403	Male	18	40.0	55	7.52	4.60	0	24
3397	Female	18	38.5	55	7.52	4.55	0.792	24
4156	Male	20	32.0	55	7.52	5.12	0.0714	24
4200	Female	12	35.4	55	7.52	4.87	5.94	24
4196	Female	12	35.5	55	7.52	4.98	0.792	24
4158	Male	11	31.3	55	7.52	5.09	0	24
4198	Female	13	31.2	55	7.52	4.77	0	24
4159	Male	12	29.5	55	7.52	4.89	0.0006	24
4160	Male	12	33.3	55	7.52	5.33	0.0072	24
4186	Female	12	34.4	55	7.52	4.83	5.94	24
4165	Male	13	31.6	55	7.52	5.15	5.94	24
4192	Female	14	36.1	55	7.52	4.68	0.0714	24
4182	Female	13	40.0	55	7.52	4.59	0	24
4188	Male	14	41.5	55	7.52	4.93	0.792	24
4195	Female	16	53.0	55	7.52	4.63	0.0072	24
4161	Male	15	49.5	55	0	0	0	24
4166	Male	15	34.2	55	7.52	5.10	5.94	24
4187	Female	15	33.0	55	7.52	5.15	0.0006	24
4189	Male	16	33.2	55	7.52	5.16	0	24
4184	Female	16	32.1	55	7.52	5.19	0.792	24
4190	Female	17	32.8	55	7.52	5.00	0.0714	24

4614	Female	13	31.8	55	7.52	5.08	5.94	24
4630	Male	14	28.2	55	7.52	5.05	0.0072	24
4633	Male	14	28.9	55	7.52	5.06	0.0006	24
4619	Female	14	31.8	55	7.52	5.28	0	24
4620	Female	17	32.2	55	7.52	4.89	0	24
4634	Male	17	35.4	55	7.52	4.95	5.94	24
4635	Male	17	36.2	55	7.52	4.97	0	24
4617	Female	17	36.5	55	7.52	5.08	0.0072	24
4631	Male	18	34.4	55	7.52	4.98	0.792	24
4615	Female	17	37.1	55	7.52	4.78	0	24
4632	Male	18	37.8	55	7.52	5.03	0.0714	24
4616	Female	18	36.2	55	7.52	5.06	5.94	24

Supplemental Figures

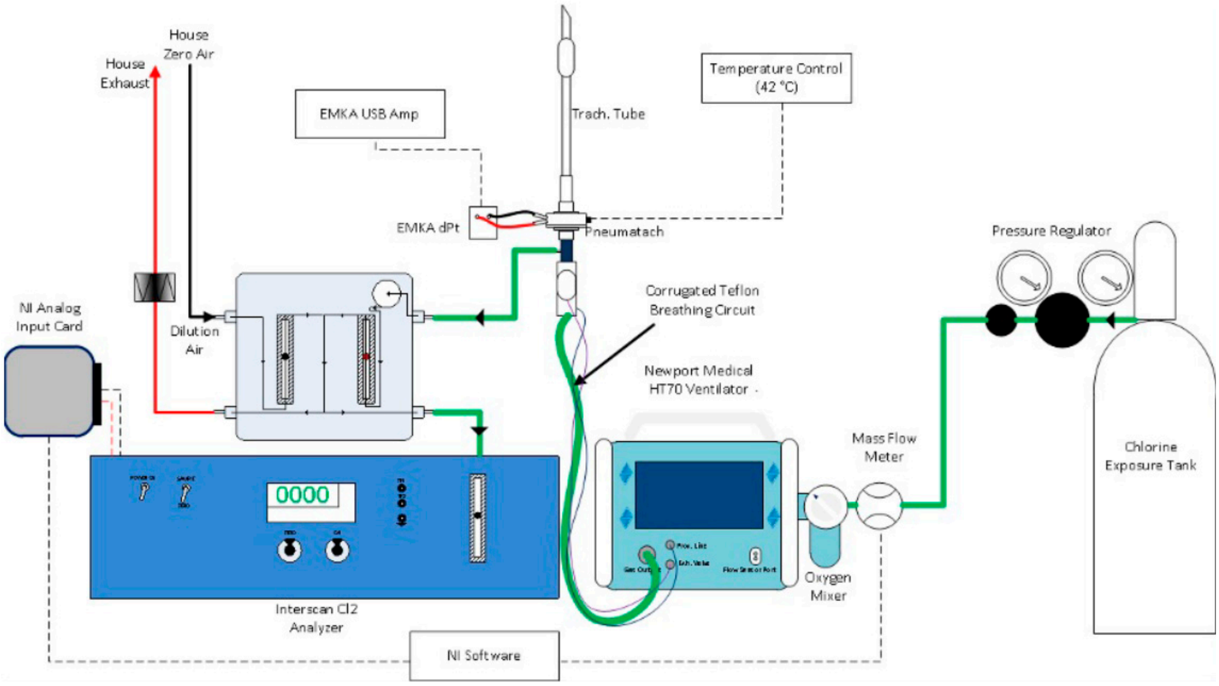

Figure S1. Chlorine exposure system schematic. Yorkshire swine were exposed to 240 ppm chlorine gas by intratracheal inhalation using volume-controlled mechanical ventilation. Chlorine gas (240 ppm balanced in medical air) was connected to a Newport HT-70 ventilator, and swine were mechanically ventilated at 12 mL/kg and 20 breaths per minute through a Teflon-lined breathing circuit. Pulmonary function and tidal volume were continuously measured throughout exposure using a pneumotachometer and pressure transducer connected to the end of the endotracheal tube.

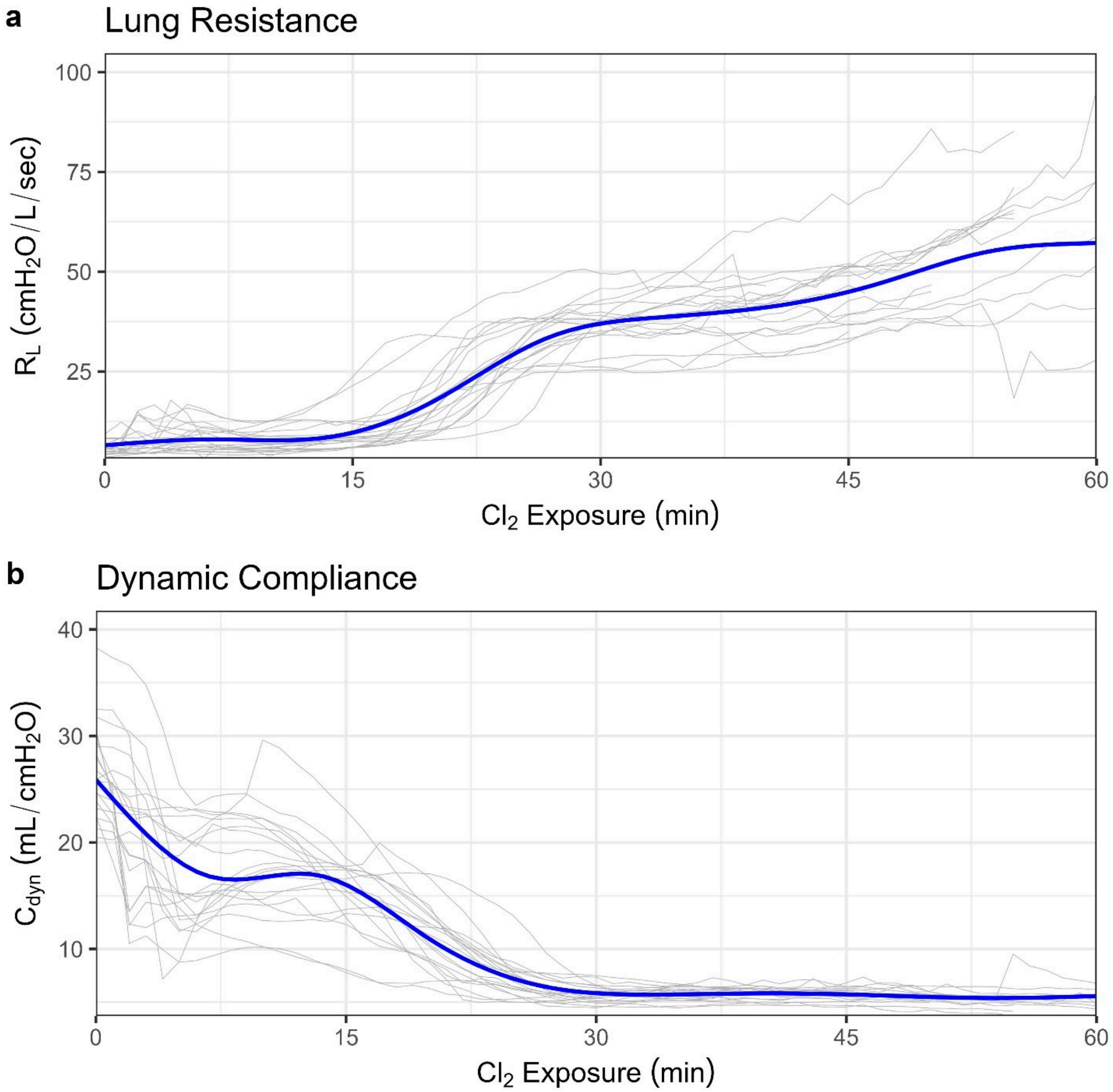

Figure S2. Kinetics of functional lung injury during chlorine exposure. Swine were exposed to 240 ppm chlorine gas (Cl_2, n=21) for between 19 and 90 minutes. Lung resistance (R_L) (**a**) and dynamic compliance (C_{dyn}) (**b**) were measured in real time during exposure from phase-matched flow and pressure signals that were integrated using EMKA iox software, and 60-second averages were calculated for each animal through the first 60 minutes of exposure. A smooth curve fit (using generalized additive models; in blue), representing the mean trajectory across all animals was traced through the individual animal trajectories for both resistance and compliance.

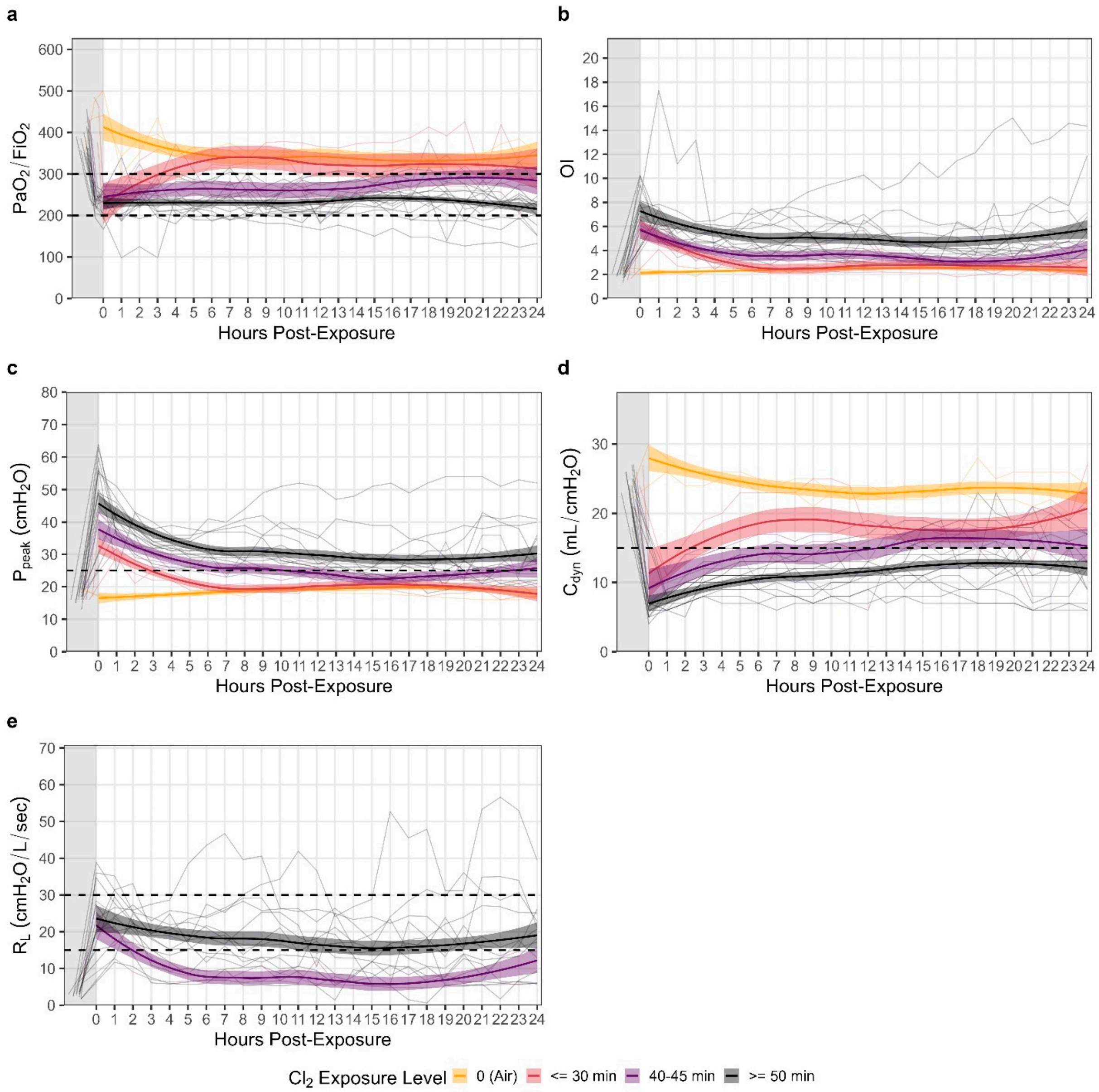

Figure S3. Kinetics of functional lung injury following chlorine exposure. Swine were exposed to 240 ppm chlorine gas (Cl_2, n=19) or room air (n=2; circles) via controlled ventilation for between 19 and 90 minutes. PaO_2/FiO_2 (P/F) ratio was calculated assuming a 21% FiO_2. The. PaO_2/FiO_2 (P/F) ratio (a), oxygenation index (OI; b), peak airway pressure (P_{peak}; c), dynamic compliance (C_{dyn}; d), and lung resistance (R_L; e) were recorded once at baseline (i.e., prior to exposure) and once hourly following the end of exposure for 24 hours (R_L only available in those animals exposed to Cl_2 for 40 minutes or greater). Dashed lines represent target thresholds for the desired acute lung injury phenotype (i.e., 200-300 for P/F ratio, 25 cmH_2O or above for P_{peak}, 15 mL/cmH2O or below for C_{dyn}, 15-30 cmH_2O/L/sec for R_L). Curve fits (and 95% confidence ribbons; obtained via generalized additive models and starting post-exposure) are presented for air controls (in yellow), chlorine exposures of less than 30 minutes (in red), chlorine exposures between 40 and 45 minutes (in purple), and exposures 50 minutes and above (in black).

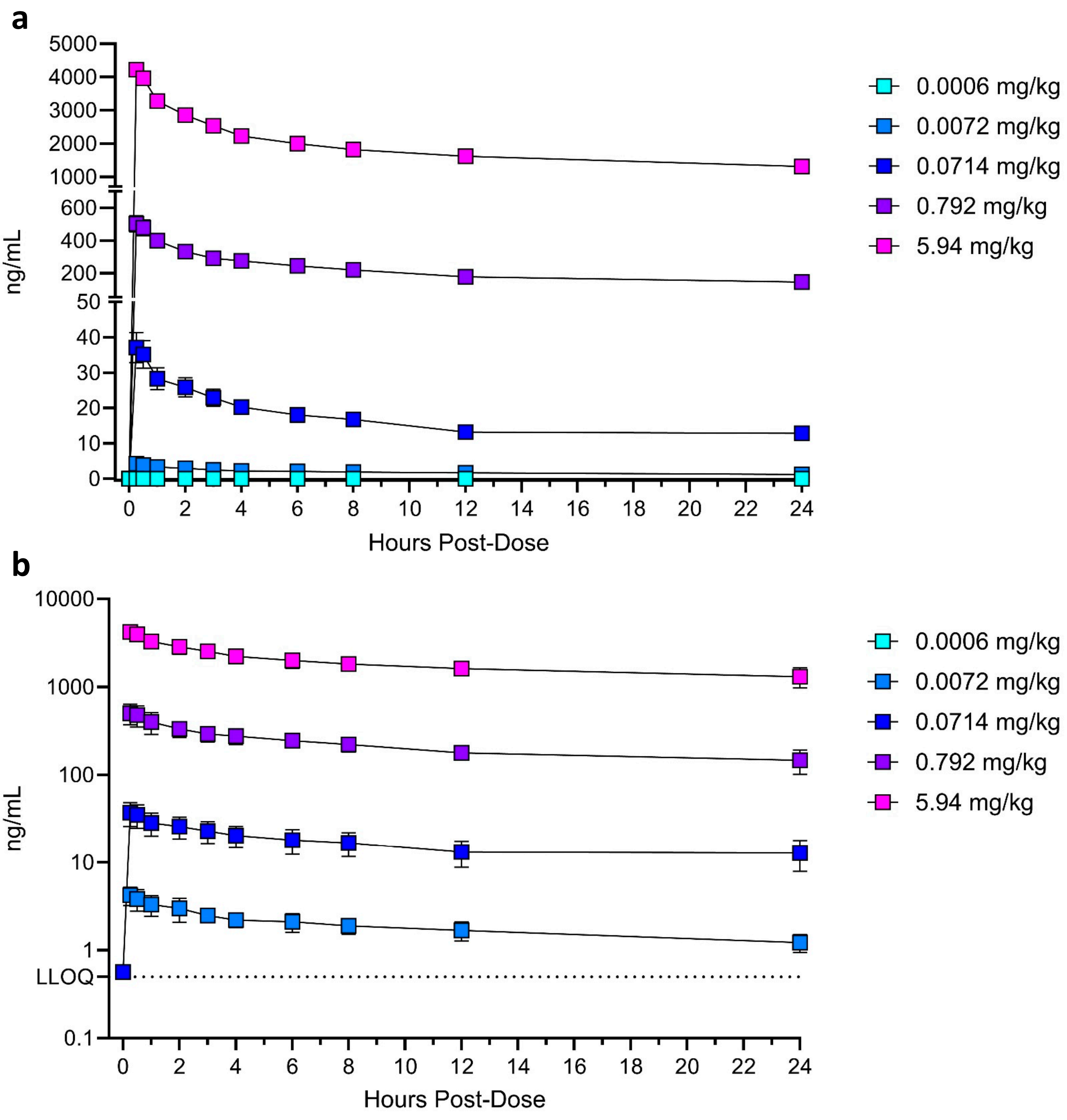

Figure S4. GSK2798745 plasma concentration vs. time profiles. Yorkshire swine were exposed to 240 ppm chlorine gas for 55 minutes. Following exposure, animals were treated with GSK2798745 by intravenous infusion (n=6-16 per treatment group). Plasma GSK2798745

concentrations, represented on (a) linear and (b) logarithmic scales, were measured by LC-MS/MS at 0.25, 0.5, 1, 2, 3, 4, 6, 8, 12 and 24 hours following the end of dosing. The mean +/- standard deviation for each experimental group are shown. Values below the assay lower limit of quantification (LLOQ) were plotted at the LLOQ (i.e., 0.5 ng/mL).

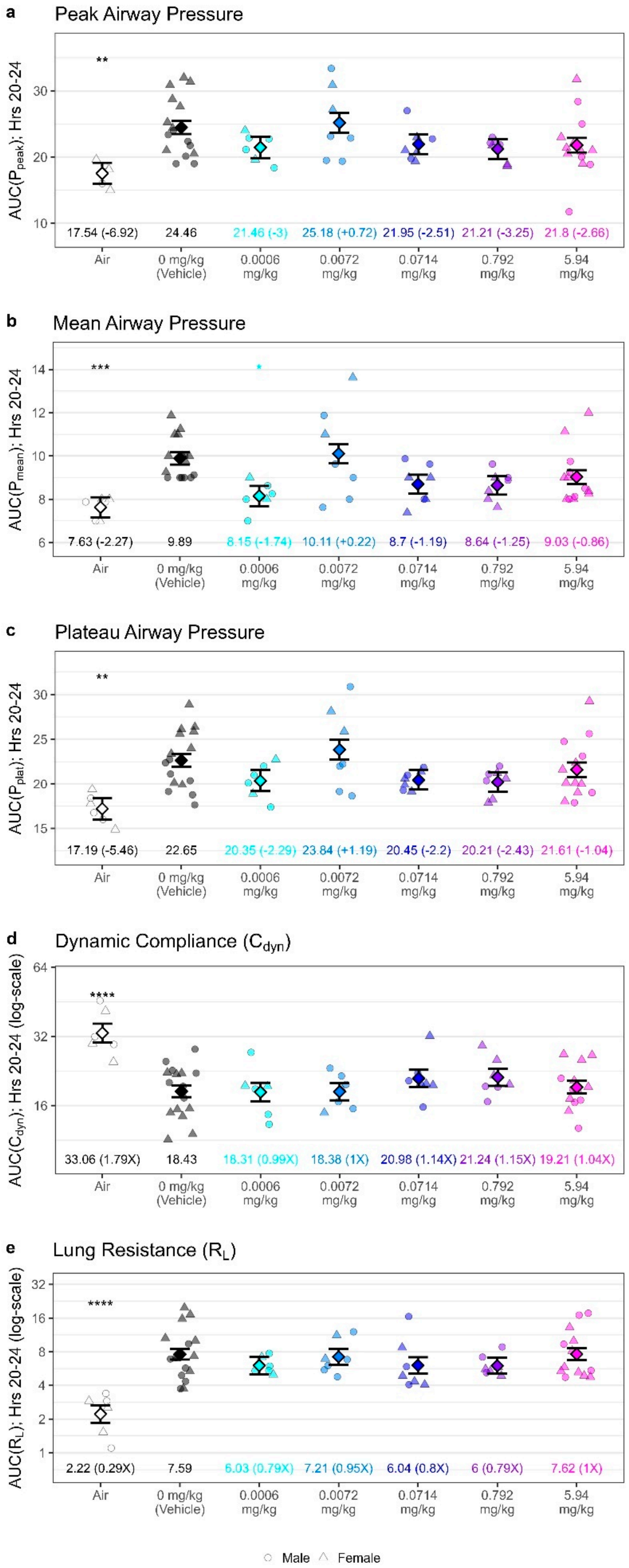
a
Peak Airway Pressure
AUC(P_peak); Hrs 20-24
**
17.54 (-6.92)
24.46
21.46 (-3)
25.18 (+0.72)
21.95 (-2.51)
21.21 (-3.25)
21.8 (-2.66)
b
Mean Airway Pressure
AUC(P_mean); Hrs 20-24
***
7.63 (-2.27)
9.89
8.15 (-1.74)
10.11 (+0.22)
8.7 (-1.19)
8.64 (-1.25)
9.03 (-0.86)
c
Plateau Airway Pressure
AUC(P_plat); Hrs 20-24
**
17.19 (-5.46)
22.65
20.35 (-2.29)
23.84 (+1.19)
20.45 (-2.2)
20.21 (-2.43)
21.61 (-1.04)
d
Dynamic Compliance (C_dyn)
AUC(C_dyn); Hrs 20-24 (log-scale)
****
33.06 (1.79X)
18.43
18.31 (0.99X)
18.38 (1X)
20.98 (1.14X)
21.24 (1.15X)
19.21 (1.04X)
e
Lung Resistance (R_L)
AUC(R_L); Hrs 20-24 (log-scale)
****
2.22 (0.29X)
7.59
6.03 (0.79X)
7.21 (0.95X)
6.04 (0.8X)
6 (0.79X)
7.62 (1X)
Air
0 mg/kg (Vehicle)
0.0006 mg/kg
0.0072 mg/kg
0.0714 mg/kg
0.792 mg/kg
5.94 mg/kg
Male
Female

Figure S5. Functional lung measurements unaffected by GSK2798745 treatment. Yorkshire swine were exposed to either 240 ppm chlorine gas or room air by intratracheal inhalation for 55 minutes. Following exposure, animals were treated with either vehicle or GSK2798745 by intravenous infusion (n=6-16 per treatment group). Peak (P_{peak}, **a**), mean (P_{mean}, **b**) and plateau (P_{plat}, **c**) pulmonary pressure, as well as dynamic compliance (C_{dyn}, **d**) and lung resistance (R_L, **e**) was measured hourly post-exposure for 24 hours, and the area under the curve (AUC) for measurements from 20-24 hours post-exposure are presented. Individual data points (jittered horizontally to remove overlap), overlayed with model estimates (diamonds) and standard errors are displayed for each experimental group, with the levels and difference compared to vehicle control listed underneath. Between-treatment comparisons to Vehicle: ** = $p < 0.01$, *** = $p < 0.001$, **** = $p < 0.0001$.
